# Supplementary material for: The interactome of intact mitochondria by cross-linking mass spectrometry provides evidence for coexisting respiratory supercomplexes
Source: Mol Cell Proteomics. 2017 Dec 8;17(2):216–32. doi: 10.1074/mcp.RA117.000470 (PMC5795388; doi:10.1074/mcp.RA117.000470)
Supplement: Supplemental Data [file supp_RA117.000470_133922_0_supp_23978_fzffwf.zip › spectra_annotation/mito_spectra_annotation/228-1-8-1-7-1.pdf]

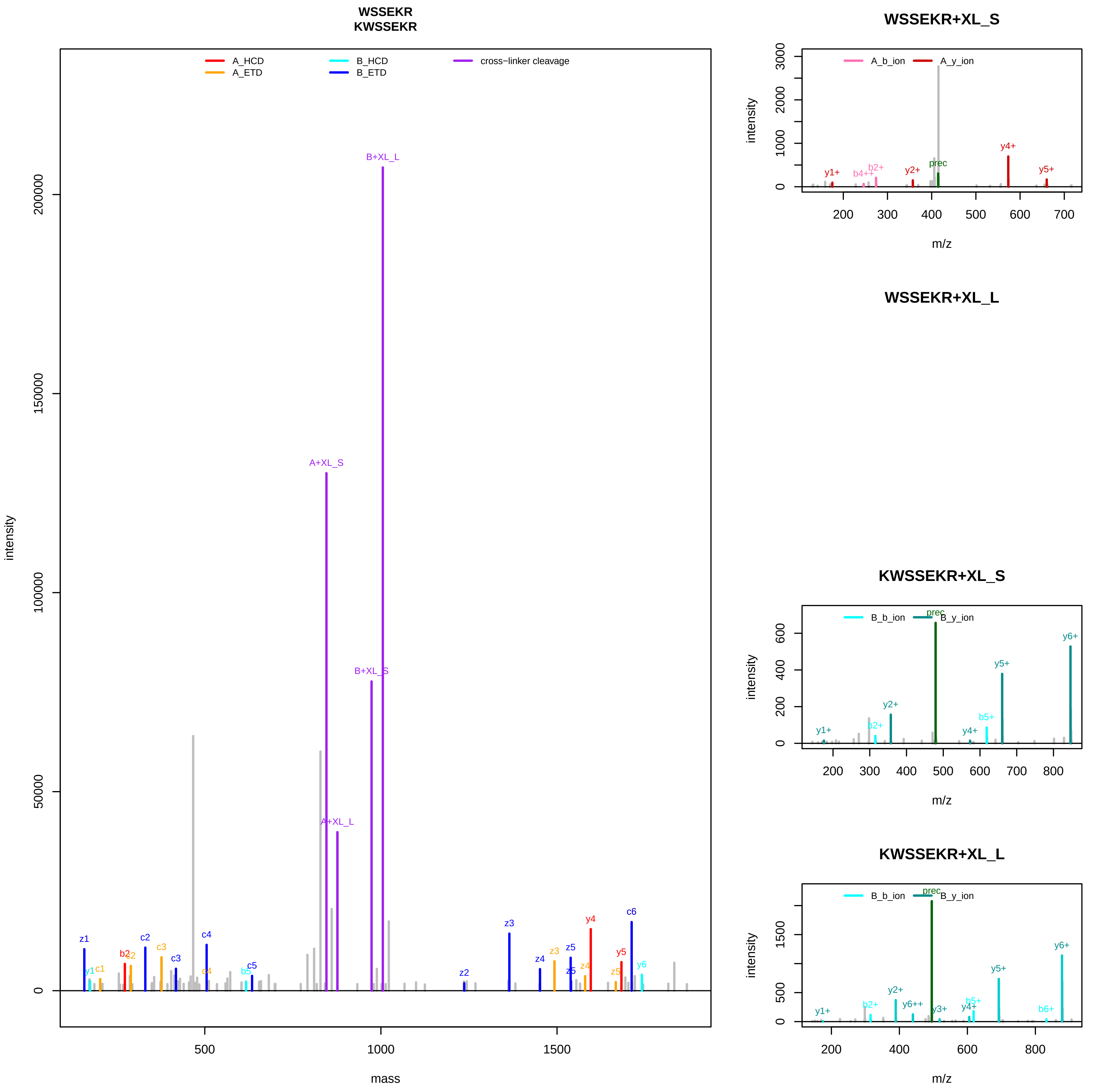

WSSEKR
KWSSEKR
A_HCD
A_ETD
B_HCD
B_ETD
cross−linker cleavage
intensity
mass
B+XL_L
A+XL_S
B+XL_S
A+XL_L
z1 y1 c1 b2 c2 c2 c3 c3 c4 c4 b5 c5 z2 z3 z4 z3 z5 z5 z4 y4 z5 y5 c6 y6
WSSEKR+XL_S
A_b_ion
A_y_ion
y1+ b4++ b2+ y2+ prec y4+ y5+
m/z
WSSEKR+XL_L
KWSSEKR+XL_S
B_b_ion
B_y_ion
prec
y1+ b2+ y2+ y4+ b5+ y5+ y6+
KWSSEKR+XL_L
y1+ b2+ y2+ y6++ y3+ y4+ b5+ y5+ b6+ y6+
